# The Good, the Bad and the Recovery in an Assisted Migration

**DOI:** 10.1371/journal.pone.0014160

**Published:** 2010-11-30

**Authors:** Bridget S. Green, Caleb Gardner, Adrian Linnane, Peter J. Hawthorne

**Affiliations:** 1 Tasmanian Aquaculture and Fisheries Institute, University of Tasmania, Hobart, Tasmania, Australia; 2 Aquatic Science, South Australian Research and Development Institute, Henley Beach, South Australia, Australia; University of Glamorgan, United Kingdom

## Abstract

**Background:**

Assisted migration or translocation of species to ameliorate effects of habitat loss or changing environment is currently under scrutiny as a conservation tool. A large scale experiment of assisted migration over hundreds of kilometres was tested on a morph from a commercial fishery of southern rock lobster *Jasus edwardsii*, to enhance depleted populations, improve the yield and sustainability of the fishery, and test resilience to a changing climate.

**Methodology and Principal Findings:**

Approximately 10,000 lower-valued, pale-coloured lobsters were moved from deep water to inshore sites (2 in Tasmania [TAS] and 2 in South Australia [SA]) where the high-value, red morph occurs. In TAS this was a northwards movement of 1° latitude. Growth was measured only in TAS lobsters, and reproductive status was recorded in lobsters from all locations. Pale females (TAS) grew 4 times faster than resident pale lobsters from the original site and twice as fast as red lobsters at their new location. Approximately 30% of translocated pale lobsters deferred reproduction for one year after release (SA and TAS), and grew around 1 mm yr^−1^ less compared to translocated pale lobsters that did not defer reproduction. In spite of this stress response to translocation, females that deferred reproduction still grew 2–6 mm yr^−1^ more than lobsters at the source site. Lobsters have isometric growth whereby volume increases as a cube of length. Consequently despite the one-year hiatus in reproduction, increased growth increases fecundity of translocated lobsters, as the increase in size provided a larger volume for producing and incubating eggs in future years.

**Conclusions and Significance:**

Assisted migration improved egg production and growth, despite a temporary stress response, and offers a tool to improve the production, sustainability and resilience of the fishery.

## Introduction

Assisted migration is the mediated movement of animals to facilitate or mimic range expansion [1], or enhance depleted populations [2]. Assisted migration can also replenish extinct or endangered populations, create arks for species under threat and help ameliorate loss of habitat due to climate change [2], [3]. While the definitions vary widely throughout the literature, assisted migration is a recent incarnation of ‘translocation’, that has been one of the most commonly used tools for biodiversity restoration worldwide [4]. Assisted migration generally involves translocating species beyond their existing or former range, to a contiguous environment where barriers to dispersal have occurred through habitat fragmentation, or to an adjacent environment with different temperature regimes [5], [6], [7].

Assisted migration is currently under debate for its value and utility as a conservation tool [8], [9], [10], [11]. Particularly, recent focus has been the potential negative impacts of shifting an animal into a new habitat, including that transplants must not displace residents, negatively impact the environment, transfer disease, or suffer negative consequences themselves [2], [3], [12]. Opinion is also divided around whether in managing a resource or ecosystem, humans should intervene any further as some past interventions, introductions and assisted migrations, such as cane toads, foxes and weevils have been catastrophic [7], [8], [13]. In addition, it is difficult to acquire the breadth of knowledge of a species and its interactions with its habitat required to accurately assess risks involved in transplanting to a new environment [11]. Positive results from assisted migration have received less attention, although translocations have been used for successful reintroductions or for the purpose of replenishment for decades [4]. For example, European lobster, *Homarus gammarus* are being enhanced and re-populated after overfishing [14], the Australian honeysuckle, *Lambertia orbifolia* was translocated to new sites to protect it from fungal infections [15], and there is a fossil record of cultivation of plants far from their natural ranges dating back thousands of years [16]. Modern horticulture, agriculture and aquaculture are responsible for wide scale translocations, within continents and ocean basins [17], [18]. The establishment of plants beyond their native range has enhanced agriculture and associated economies [18], and some marine harvest industries are following similar pathways to these terrestrial examples for growth, replenishment and restoration.

Recently, assisted migration has been used when a species or habitat is under threat by direct anthropogenic impact or climate change, and involves a pole-ward movement to adjacent environments with lower temperatures [7]. In the present experiment we test its utility to increase fishery production, value and build resilience to climate change in a productive and well managed fishery, the southern rock lobster, *Jasus edwardsii*. Secondarily, instead of pole-ward movement for climate change refuges we have included an equator-ward movement to enable us to predict climate change effects on the fishery and assess potential climate change risks to the fishery. We tested the effect of moving lobsters northward to climates they are predicted to encounter under mid-range future climate change scenarios [19], [20]. This fishery has special interest in the context of climate change because it is located in one of the fastest warming areas of the globe and is exhibiting declines in recruitment in multiple regions that are consistent with climate change [19], [21]. Despite an overall healthy biomass, the southern rock lobster fishery is characterised by spatial heterogeneity in demographic traits and some local depletion through fishing [22]. *J. edwardsii* from deep water, offshore sites generally have slower growth, earlier maturation, and paler colouration than rock lobster from warmer, shallow water [23], [24], [25]. This variation in demographic traits influences the yield and the economic drivers of the fishery. After the introduction of a quota cap (TAC) and individual transferable quota's (ITQ's) in 1996, fishers could no longer increase revenue by increasing their catch, so they maximised the value of their catch by targeting higher value red lobsters that inhabit inshore areas [23]. This coupling of large-scale spatial heterogeneity in a natural resource and economic incentive to maximise the return on ITQ investment has led to localised depletion of inshore stocks [22], [26]. Recreational fisheries have also expanded in inshore areas and the combined effect of all sectors appears also to have had a cascading ecological effect via a phase shift, from a population explosion of an urchin species *Centrostephanus rogersii* [27].

In line with emerging theories on managing socio-economic resilience [28], we examined assisted migration within a healthy and productive fishery as a tool to increase stock resilience to frequent human and natural perturbations. We translocated the pale, low value morph of a key fisheries species to a range of latitudes and depths that are representative of future temperatures under climate change predictions [19], [20]. The objectives were to test assisted migration to increase the value and biomass of a marine stock while reducing negative effects of fishing pressure; and to understand the effects of increasing temperature on a valuable fishery, with the overall goal of increasing resilience to perturbations such as climate change.

## Materials and Methods

### Southern rock lobster

All handling of rock lobster in this study met the Australian Government National Health and Medical Research Council code of practice for the care and use of animals for scientific purposes. Although currently ethics approval is not required for research on invertebrates under this code of practice, the guidelines for ethical and humane treatment of animals in research were followed in all handling of lobsters.

*Jasus edwardsii* is an exploited iteroparous spiny lobster with indeterminate growth, which inhabits temperate rocky reefs throughout southern Australia and New Zealand. *J. edwardsii* are relatively site attached [29], [30] with a pelagic larval phase and possible mixing of stocks through larval dispersal [31], [32]. Size of maturation varies along with growth and other demographic traits throughout their range [33], [34], [35], [36].

### Study sites

**Figure 1:**
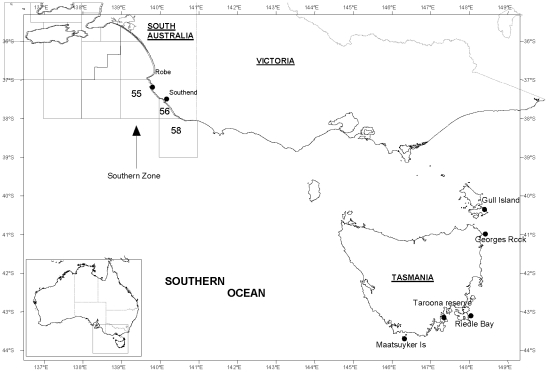
Map of study sites in South Australia and Tasmania. Inset is map of Australia.

This experiment was undertaken in two fishery jurisdictions: Tasmania (TAS) and South Australia (SA), Australia. A total of five sites were used in the study: the TAS source site (Maatsuyker Island); two TAS transplant sites (Riedle Bay and Taroona Reserve); one offshore SA source site within Marine Fishing Area (MFA) 55 of the SA southern zone rock lobster fishery; and two SA transplant sites near Southend (37°34′090′S, 140°07′504′E) in MFA 56 and Robe (38°03′398′S, 140°41′949′E) in MFA 56 (Fig. 1). Maatsuyker Island (43°40′30″S 146°12′56″E) is a southern, deep-water (60–100 m) rocky reef 12 nm offshore where lobsters grow slowly, are pale in colour [24], size at first reproduction (SOM) is small at around 60 mm carapace length (CL) and females rarely reach the minimum legal size of 105 mm (CL) [36]. Mean summer SST for Maatsuyker Is. ranges from 12–14°C, and is predicted to increase to 14–17°C in 2030 and 17–19°C in 2070 under mid-range IPCC scenarios [19], [20]. Taroona Reserve (42°57′08″S 142°21′20″E) and Riedle Bay (42°40′09″S 148°6′13″E) are shallow rocky reefs approximately 1° latitude north of the source site, where lobsters grow faster and are red in colour [24]. Taroona Reserve is a shallow estuarine rocky reef bounded by large expanses of sand, with depth 7–15 m. The area is approximately 1.24 km^2^ including a surrounding no-take buffer zone. Population density of *J. edwardsii* within the reserve is high (approximately 13 000 individuals), and the reserve has been closed to both commercial and recreational fishing since 10 November 1971 when it was proclaimed as a marine reserve for rock lobster research. Riedle Bay is an inshore exposed rocky reef of granite boulders and macroalgae, with continuous reef to 60 m. The average depth is 15 m. Mean summer SST for both Taroona and Riedle Bay is 15–17°C [19].

The offshore South Australian site where lobsters were caught for translocation was located at in MFA 55 at a depth of ∼100 m. Catch rates within this area have been historically high (up to 3 kg/potlift) which presumably reflects low fishing effort in offshore regions of the fishery [26]. The SOM at this site was ∼68 mm CL [34]. The inshore SA sites at Southend and Robe to which lobsters were translocated were broadly similar consisting of limestone reef matrices, eroded to form ledges, crevices, undercuts and holes. Both were located in 15–20 m depth. Pre-site selection dive surveys indicated that the reefs were dominated by encrusting invertebrates (sponges, ascidians, bryozoans), spiny urchins (*Heliocidaris erythrogramma*), red foliose, green foliose (*Caulerpa sp.*), brown branching (*Ecklonia radiata*, *Macrocystis angustofolia*), and encrusting coralline algae. The SOM in the region was ∼93 mm CL [35].

### Translocation

Translocations occurred at different times in the two fisheries. In Tasmania, in the austral summer from 2005 to 2007, 5747 undersized mature female lobsters were captured from Maautsuyker Is. and moved to the two experimental sites. Three translocations occurred from Maatsuyker to Taroona and one to Riedle Bay Lobsters were caught using 50 metal mesh lobster pots baited with a barracouta head (*Thyrsites atun*) and a jack mackerel (*Trachurus declivis*) and deployed in an area 500 m×120 m. Pots were emptied twice daily, once at daybreak, then redeployed and emptied again after midday. At capture, *J. edwardsii* were measured and tagged on the ventral surface of the first or second segment of the abdomen with a uniquely coded t-bar tag (Hallmark, Victor Harbour, South Australia). All rock lobsters for translocation were immediately placed in 2×4000 l flow-through tanks onboard the RV “Challenger” under ambient water conditions where they were held until release at the new location (2–3 d). Lobsters were released at the water surface into an 80-m diameter net, height 1.5 m with braided nylon mesh (stretched mesh size of 21 mm diagonal) set on the sea floor in the Taroona Reserve and at Riedle Bay for 24 h to reduce their initial flight response away from the release site [37] and prevent any subsequent predator mortality upon release [38]. The base of the net was open and weighted by chains, and the walls were suspended by foam floats. The cage was roofless. Release of translocated lobsters onto the reef within the cage occurred at night-time to reduce predator mortality. After 24 h, the cage was lifted and the lobsters were then free to move around the reef. No mortality was observed within the cage or by predators upon release. Lobsters caught and released at the capture site were tagged and measured handled in the same way, but were released immediately after tagging without storage in flow-through tanks and transport. Six lobsters died during transport to Taroona in 2008, and 17 in SA throughout the 2 translocation trips. No other mortalities were observed.

In South Australia in summer of 2007, 4589 lobsters, including 3073 females (441 immature, 2632 mature) were captured over two trips, using the same gear as described for TAS. Lobsters were transported in 2×3000 l flow-through tanks on board a commercial fishing vessel chartered for the operation. Lobsters were in transit for 2–8 h from the time of capture until they were released late afternoon onto highly complex limestone habitat chosen for its availability of crevices and refuges at the release site. The release sites were highly exposed with large swell, and these conditions did not allow for a net to be set up for acclimation and initial protection.

### Sampling at transplant sites

TAS. In total there were 13 sampling trips at Taroona and 3 at Riedle after the release of the translocated lobsters. Sampling took place bi-monthly in 2006 from January, 3 times a year in 2007 and 2008 and once in 2009 at Taroona. Sampling at Riedle occurred in December 2007, July 2008 and April 2009. All sampling used the same capture method as for the translocated lobsters described above. Pots were set for three days at each site, and checked each morning and redeployed. All lobsters were measured for carapace length (CL), checked for a tag, gender, maturational status, and berried status.

SA. In Oct-May 2008/09 sampling for females occurred through two designated trips using the methods described above, although most of the data on reproduction was collected through a commercial tag return program [34].

### Grading maturational status and measuring growth

*J. edwardsii* females mate in the austral autumn, and females brood their eggs on their abdomen for 3–4 months. Eggs are attached to the endopodite processes on pleopods by ovigerous setae. The presence of ovigerous setae was used as an indication of maturity [39], and their absence indicated that animals were incapable of carrying eggs on the abdomen. All lobsters collected from Maatsuyker Is. were observed to have mature setose pleopods when transplanted, so any record of absence of setae was an indication of deferred or regressed reproduction. At each recapture, lobsters size (CL) was measured. Growth per year was determined by comparing the size at last recapture to the size at tagging, and dividing it by years at liberty, with fractions of years adjusted for the likelihood of a moult occurring. Lobsters grow in increments at each moult, males moult between September and October and females in April and May. Therefore growth (g) per year was calculated as g = s^t2^ – s^t1^/T (yr), where s =  size at sampling time (t), and T is time in years.

### Analyses

In SA there was no transplant control and growth measures were not be taken from residents due to constrained resources, so comparison of growth at source and translocated sites was made on TAS *J. edwardsii* only. An ANCOVAof growth comparing lobsters of different maturational status were performed on females from TAS (Taroona Reserve, Riedle Bay) and SA (Robe and Southend) one year after translocation, with initial size as a covariate.

## Results

**Figure 2:**
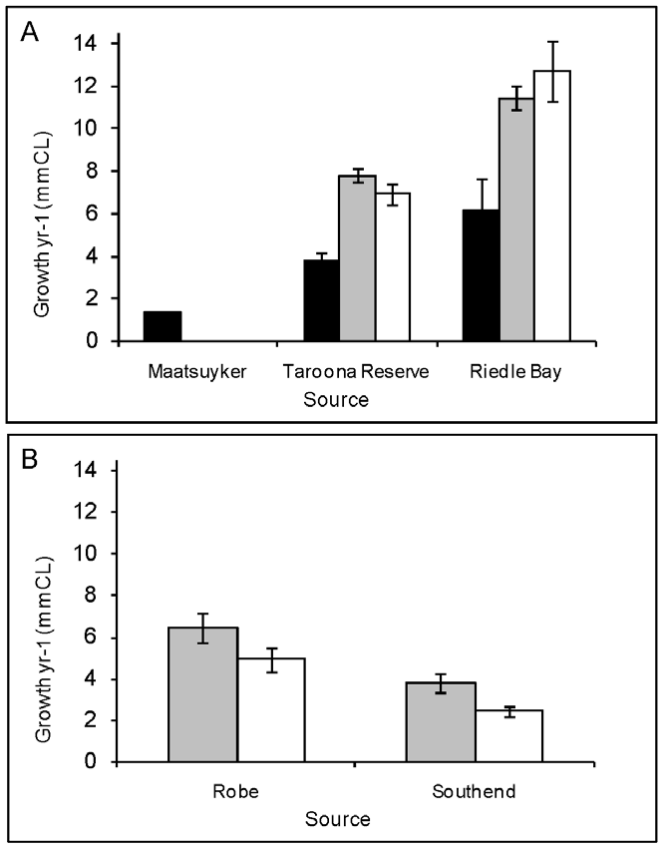
Growth rate of *J. edwardsii* females. Growth rate of females that remained mature or regressed after translocation, compared to growth of resident lobsters at the translocation sites Growth of resident and translocated lobsters measured 2 years after translocation. a. TAS, b. SA 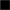
Residents, 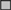 Translocated lobsters which maintained reproduction, 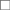
Translocated lobsters which deferred reproduction. Error bars are SE.

**Figure 3:**
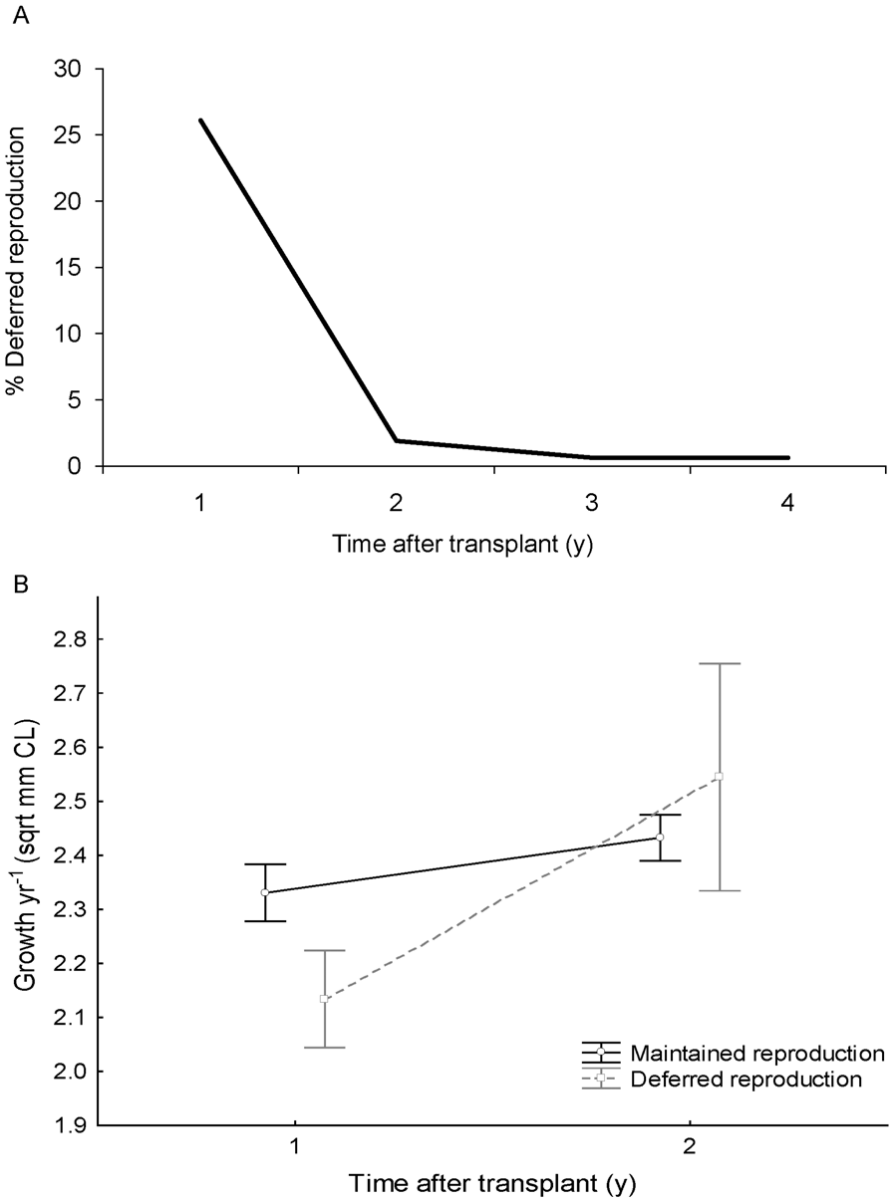
Females with deferred reproduction. a. Percentage of female *J. edwardsii* that deferred reproduction over 4 years of sampling at Taroona Reserve. b. Growth rate of translocated lobsters that deferred reproduction or maintained reproduction for two years after translocation.

**Figure 4:**
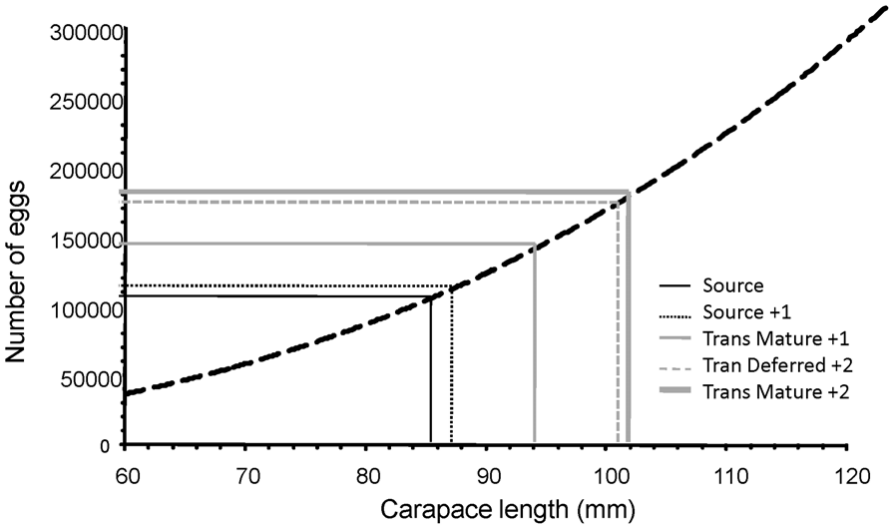
Estimated change in annual fecundity after translocation. Estimation change in annual fecundity after translocation for an 86 mm *J. edwardsii*, from on length-fecundity estimates from Green et al [33]. “Source” is Maatsuyker island, +1 is after 1 year and +2 is after two years. Mature refers to lobsters that maintained reproduction and ‘deferred’ refers to the 30% that deferred reproduction for 1 year.

External changes to accessory reproductive limbs occurred within one moult in pale females after the assisted migration to the new habitat. Approximately 30% of translocated pale female lobsters deferred reproduction for the first year in their new habitat by changing the structure of their pleopods at moult and producing endopodites without ovigerous setae. The exact proportion of females that deferred reproduction varied with site: of the 157 female transplants recaptured at Taroona, 26% of females deferred reproduction, and in SA between 32 and 36% of females ceased reproducing (Table 1). For lobsters translocated within Tasmania, growth of all translocated lobster exceeded growth of resident lobsters at the sites to which they were translocated by 3 to 6 mm yr^−1^ in the first year after translocation (Fig. 2a, Table 2), and was more than four times the growth rate of lobsters at the source site Maatsuyker island, 6.4±0.5 mm c.f 1.4±0.06 mm yr^−1^ (F_1,362_ = 121, p<0.0005). Growth rate of lobsters that deferred reproduction was reduced at 3 of the 4 translocation sites, and remained the same at one site (Riedle Bay, TAS, Fig. 2a, b, Table 2), compared to translocated lobsters that maintained maturity, but was still significantly higher than growth rate at the source site. Two years after translocation 93% of females that had become non-reproductive had recommenced reproduction (Fig 3a), and growth of lobsters that had regressed reproduction matched growth of translocated lobsters that had continued reproduction (Fig. 3b). Despite 30% of females suspending reproduction for a season, estimated egg production improved with translocation by approximately 35,000 eggs yr^−1^ for translocated individuals beyond what would have occurred after one year at the source site. This is because lobsters that deferred reproduction for 1 yr egg production increased egg production by 65,000 eggs yr^−1^ in the second year after translocation, and lobsters that remained mature increased egg production by approximately 80,000 eggs yr ^−1^ (Fig 4).

**Table 1:** Sampling.

Area	% deferred reproduction	Number recaptured
**TAS**		
Riedle Bay	20.5	78
Taroona	26	157
Maatsuyker Island	0	531
**SA**		
Robe	32.5	80
Southend	36	150

Summary of maturational status of female rock lobster recaptured 1 year after translocation from deep to shallow water.

**Table 2:** Summary of ANCOVA of *J. edwardsii* growth at sites of translocation in the first year after translocation.

	DF	MS	F	p
Carapace length	1	942	63	<0.005
Site	3	327	21	<0.005
Maturation status	1	74	5	<0.05
Site*Mat status	3	8	0.5	0.655
Error	381	14		

Length was covariate.

## Discussion

This transplant experiment demonstrated a complex response in reproduction and growth in *Jasus edwardsii* to assisted migration which varied throughout the range of the species. In popular parlance these results can be summarised as “the good, the bad and the recovery” of demographic traits following an assisted migration. Increased growth in the new location was a positive outcome in terms of the goals of this assisted migration, offering a method of increasing the biomass of the stock independent of recruitment, and demonstrating increased growth of cold water, pale lobsters when shifted to a warmer environment. Faster growth of translocated lobsters increases the average weight of lobsters which in turn would reduce the total number of lobsters harvested by the commercial sector under ITQ management. On the negative side, reproduction was deferred in approximately 30% of females for one year, which reduced the fecundity of the population for that year, and is a possible indicator of stress resulting from the translocation. Encouragingly, there was recovery of reproductive output so that the majority of females became reproductively active in the second year after release. These had maintained significantly higher growth rates compared to lobsters from both their site of origin and the residents of the translocation site. The increased growth for all females that occurred during this time would result in a total higher egg output, increasing the resilience of the fishery by improving population fecundity.

### The good: Improved fishery productivity and inherent resilience to climate change

Translocated lobsters increased growth rate to more than double that of the residents in the area to which they were moved and four times that of the lobsters in their original location. Increased growth rate, coupled with a colour change to the more valuable red colour within 12 months of translocation [24] and survival comparable to residents [40] increases the biomass and value of the lobsters available to the fishery. This increase in stock occurred in inshore areas that are those most impacted currently in response to economic processes and overfishing [23], [41]. Increased inshore productivity and larger average size could reduce ecological effects of fishing, as localised depletion coupled with climate change is the source of devastating phase shifts [27].

Equally importantly, the growth increase after the northward translocation of the southern morph denotes plasticity in response to mid-range climate change scenarios [19], [20]. While an increase in growth rate of an ectotherm is not an unexpected result following an increase in temperature [42], it does demonstrate an inherent plasticity within *J. edwardsii* populations to a range of temperature regimes and to a short-term change in temperature, offering a positive contrast to the many gloomy climate change forecasts [43], [44].

It is impossible to disentangle the physiological cause of the increased growth rate observed in this assisted migration experiment. A change in temperature is obviously not the only change faced by translocated lobsters in their new habitat. The change in location would have encompassed a change in predator and prey fields, including prey type, abundance and quality, and also changes in available and type of refuges. The change in prey types was apparent in a change in nutritional quality of translocated lobsters. One year after translocation the level of omega-3 fatty acids had increased in translocated lobsters, beyond the level of residents in the new habitat, reflecting a change in food quality [45]. Furthermore, the change from the pale white colour to the deep red colour after translocation [24] reflects an increase carotenoids, and the key source of these in marine ecosystems is astaxanthin found in green algae, present only in shallow water. A parallel study determined that foraging and home range overlapped between residents and translocated lobsters, suggesting that refuge availability was not limiting (B.S Green unpub. data).

An increased growth rate above that of the locals under the same temperature, food and prey regime is most notable, indicating that not all *J. edwardsii* are growing at the maximum possible in their environment. It is an ongoing curiosity in ecology that most animals do not grow at their maximum rate [46], [47] given the importance of somatic growth rates to fitness and survival [48], [49]. Assisted migration offers a way of moderating human impacts on local marine ecosystems, sustaining the delivery of harvestable resources, and understanding potential changes to sectors of the fishery under changing temperatures and environment.

### The bad: Skipped spawning a response to stress?

Deferred reproduction occurred at all four translocation sites in approximately the same proportions. Skipped spawning is uncommon in rock lobsters and the recorded change in maturational status based on pleopod morphology was unambiguous. In contrast there has been no observed change in pleopod morphology of animals in the control site sampled in this study or in the 28,700 lobster recaptures analysed in previous research of the Tasmanian *J. edwardsii* population (Gardner et al., 2005). This suggests that skipped spawning in 30% of females recorded after assisted migration is not part of a normal strategy to increase lifetime fecundity by optimising energy allocation [50], [51], [52], rather it appears to be a stress response to one or more of the elements of assisted migration. Animals with accessory reproductive costs such as migration or brooding (for example *J. edwardsii*), are expected to have a higher incidence of skipped spawning as they optimise energy allocation to reproduction and accessory reproductive activities [52]. When there are conflicts in the allocation of energy, a trade-off between competing processes can occur [53]. Prior to first maturation, this trade-off occurs between growth and the physiological changes required to produce gametes or secondary sexual characteristics or behaviours in order to attract a mate. After maturation, individuals repeatedly select between allocation of energy to reproduction or maintenance [50], and despite being physiologically equipped for reproduction and having the potential to spawn, many individuals will divert energy into other processes such as growth, maintenance or a stress response to maximise their fitness [51]. Skipped spawning has been documented in animals such as birds and fish frequently when conditions are poor [52], [54], [55], [56] and rarely when conditions are good [57], [58]. The potential causes of the stress response in this case include transport, increased temperature at the new site, disorientation upon release, or unfamiliar habitat. Capture and tagging can be ruled out due to the absence of any previously recorded hiatus in reproduction despite years of capture and tagging [39].

### The recovery: more eggs in bigger baskets

These experiments suggest there are opportunities for increasing fishery productivity and egg production by moving slow growing lobsters to sites that are more favourable to growth, and therefore egg production [22], [41]. These movements could be used to target specific management issues such as improving biomass and egg production in locally depleted areas [22], [27]. While translocating lobsters increases growth and therefore capacity for egg production, it also exposes females from the deep water to increased risk of fishing mortality as they grow to legal size more rapidly. Currently egg production in deep water areas of SW Tasmania is estimated to approximate virgin levels due to the slow growth to legal size, while at some inshore sites it is below 15% [59]. So while translocation exposes females to fishing mortality that they would otherwise avoid through size limit refuge, it also increases individual egg production and shifts egg production to areas where it was previously depleted, buttressing egg production over a wider area. Rock lobsters have a long larval phase with extensive larval mixing, and so increased egg production can enhance inshore and offshore stocks. Translocation has the potential to be used as a tool for management of the spatial distribution of egg production, plus should raise global egg production provided the TAC is not increased to remove the increased productivity from faster growth.

This study demonstrates the utility of assisted migration to improve the biomass of a stock and rebuild depleted stocks or sub-populations, but highlights that while overall growth rates improve, there are biological costs to transport and acclimation to a new habitat that were seen in deferred reproduction. Assisted migration via translocation of undersized and low value lobsters is a means to increase social-ecological resilience in southern rock lobster fisheries, which can align ecological and economic interests [28] with lower harvest of high value fish. This experiment demonstrated resilience and plasticity within the pale morph to an increase in temperature in line with climate change predictions within this area [19]. The establishment of plants beyond their native range is essential for successful agriculture and the economies built around that [18]. Similarly, assisted migration is a tool for stock enhancement in a healthy marine fishery, and would increase the degree to which the ecosystem can absorb human perturbation and regenerate from local depletions.
